# Diagnosis and treatment of 33 patients with primary melanoma of the female reproductive system

**DOI:** 10.3389/fonc.2025.1615749

**Published:** 2025-08-26

**Authors:** Yunge Gao, Jian Dong, Ligang Chen, Xin Guo, Lingling Liu, Weinan Guo, Cuiling Ma, Biliang Chen, Jian Wang, Xiaohui Lv

**Affiliations:** ^1^ Department of Obstetrics and Gynecology, Xijing Hospital, Air Force Military Medical University, Shannxi, China; ^2^ Department of Obstetrics and Gynecology, Xi’an People’s Hospital (Xi’an Fourth Hospital), Shannxi, China; ^3^ Department of Endoscopic Surgery, Air Force 986th Hospital, Air Force Military Medical University, Shannxi, China; ^4^ Department of Dermatology, Xijing Hospital, Air Force Military Medical University, Shannxi, China

**Keywords:** female reproductive system, melanoma, surgery, immunotherapy, comprehensive treatment

## Abstract

**Objective:**

This study aims to investigate the clinical and pathologic characteristics, treatment, and prognosis of primary melanoma in the female reproductive system.

**Methods:**

A retrospective analysis was performed using the clinical and pathological data of 33 patients with melanoma of the female lower genital tract who presented at the Department of Gynecology at Xijing Hospital of Air Force Military Medical University between June 2006 and July 2022. Pathological review and immunohistochemical staining were performed in all cases to confirm the diagnosis.

**Results:**

A total of 15 (45%) cases of primary melanoma involved the vulva, 16 (49%) involved the vagina, and two (6%) involved the cervix. The HMB-45 positive rate was 93%, the S-100 positive rate was 90%, and the Vim positive rate was 100%. Of these 33 cases, we had complete clinical information on 27 of them who received various comprehensive treatments including surgical treatment, postoperative radiotherapy, chemotherapy, immune therapy, or targeted therapy. Patients with stage III–IV comprised 67% of all cases, and the average duration of follow-up was 24 months. The 2-year recurrence rate was 82%, and the 3-year overall survival rate was 42%.

**Conclusions:**

The incidence of melanoma in the female reproductive system is low, and the prognosis is poor owing to the difficulty of early diagnosis and the high possibility of metastasis. Individualized comprehensive treatment like surgical treatment combined with immunotherapy can prolong the survival time and improve the prognosis of patients.

## Introduction

Primary malignant melanoma of the female reproductive system is a rare and highly malignant tumor (1), representing approximately 5% of melanoma cases in female patients (2). There have been limited reports on this condition, most of which are case reports, resulting in an insufficient understanding of the disease. The early stage of the disease is often misdiagnosed and underdiagnosed owing to the hidden location of the primary malignant melanoma in the female reproductive system and the lower public awareness. Metastasis can also easily occur owing to the rich blood supply in the female reproductive system (3, 4). The lack of a unified staging standard and treatment for melanomas in the female reproductive system may be attributed to the rarity of cases and the unique site of the disease. The staging standards used clinically by most gynecologists and dermatologists are different. In this study, we retrospectively analyzed the clinical and pathological data of 33 female patients with primary malignant melanoma of the reproductive system who were treated at the Department of Gynecology of Xijing Hospital of Air Force Military Medical University from 2006 to 2022. We discussed the clinical and pathological characteristics, treatment, and prognosis of these patients. Furthermore, we summarized the diagnostic and treatment experiences to provide further guidance for clinical practice.

## Materials and methods

### Clinical data collection

Data of 33 female patients diagnosed with melanoma in the reproductive system who presented at the Department of Gynecology of the Xijing Hospital of Air Force Military Medical University from 2006 June to 2022 July were collected. Patients with primary melanoma in other locations, patients lacking prior treatment records, and patients with other types of primary malignancies were excluded. The diagnoses were confirmed through pathological immunohistochemical staining.

The collected data included information regarding the patients’ age, initial symptoms (abnormal bleeding, abnormal discharge, or presence of a mass), tumor location, tumor size, tumor stage, results of pathological immunohistochemical analysis, surgical method used, and comprehensive treatment plan. The comprehensive treatment included postoperative radiotherapy, chemotherapy, immunotherapy (interferon α-1b/2b, PD-1, PD-L1), and targeted therapy (vascular endothelial growth factor inhibitor). The follow-up period commenced with the first treatment of the patient until the patient experienced relapse, metastasis, or death. The follow-up ended in November 2023, with an average follow-up duration of 24 (range, 1–108) months. Survival analysis was performed for 27 patients who were hospitalized and received treatment after three patients who were lost to follow-up after the initial treatment were excluded.

Overall survival (OS) was defined as the time from the initial treatment to either death or the last follow-up. Progression-free survival (PFS) was defined as the time from the initial treatment to relapse or the last follow-up. In this retrospective study, the patients were not evaluated by imaging at uniform intervals. Specifically, only two patients underwent whole-body computed tomography (CT) or magnetic resonance imaging (MRI), and only five patients had magnetic resonance imaging of the brain. As a result, an accurate assessment of systemic metastatic lesions could not be conducted. Then, data were collected from 21 patients who had chest, abdominal, and pelvic CT/MRI before and after the initial treatment. Excluding two cases of metastasis that occurred before treatment, the treatment efficacy of 19 patients was evaluated based on RECIST (Response Evaluation Criteria In Solid Tumors) v.1.1, with the pre-treatment imaging evaluation as the baseline. The detailed clinical data of 33 patients are provided in the **Supplementary Materials**.

### Statistical analysis

Data were analyzed using SPSS statistical software version 15.0 (SPSS, Inc., Chicago, IL, USA). Percentage statistics were performed for counting data. Kaplan–Meier survival curves were drawn using the R package survival.

## Results

### Clinical information

The 33 patients ranged from ages 34 to 74 years old, with a median age of 59 years. Melanoma of the reproductive system was more common in postmenopausal women (26/33, 79%). The most common initial symptom was abnormal masses appearing in the vulva, vagina, and cervix (18/33, 55%), followed by abnormal bleeding (13/33, 39%). Of these 33 patients, five patients presented with lesions involving both the vagina and either the cervix or vulva at initial diagnosis. The primary lesion determined by histopathology was used to classify the site of onset. Accordingly, 15 (45%) cases involved the vulva, 16 (49%) involved the vagina, and two (6%) involved the cervix. Of the 33 patients, 30 patients were hospitalized and received treatment, whereas three patients were lost to follow-up after the first hospitalization. Moreover, three patients were diagnosed only through biopsy at the outpatient clinic and did not receive further treatment. The age, site of tumor occurrence, and immunohistochemical results of all patients are presented in **Table 1**. The clinical data for patients are provided in **Table 2**.

**Table 1:** Clinicopathological features of primary melanoma in female reproductive system (n=33).

Feature	n%
Age (Years)	
<50	6(18%)
≥50	27(82%)
Anatomic site	
Vulva	17(52%)
Vagina	18(55%)
Cervix	3(9%)
Histologic subtype (n=19)	
Superficial spreading	8/19(42%)
Nodular	11/19(58%)
Positive rate of immunohistochemistry	
HMB-45(n=30)	28/30(93%)
S-100(n=29)	26/29(90%)
Vimenti(n=15)	15/15(100%)
Ki-67(n=29)	
<30%	7/29(24%)
30-60%	19/29(66%)
>60%	3/29(10%)
Vascular invasion (n=14)	
Positive	2/14(14%)
Negative	12/14(86%)

**Table 2:** Clinical data of female reproductive system melanoma patients (n=33).

Characteristic	n%
Initial symptom	
Abnormal bleeding	13(39%)
Abnormal tumor	18(55%)
Abnormal discharge	1(3%)
Pigmentation	1(3%)
Course of disease	
<3 month	17(52%)
3-6 month	8(24%)
>6 month	8(24%)
Menopause	
Yes	26(79%)
No	7(21%)
Family history of tumor(n=29)	
Yes	4(14%)
No	25(86%)
Tumor diameter(n=28)	
<3cm	14(50%)
≥3cm	14(50%)
Pigmentation	
Yes	31(94%)
No	2(6%)
Color of lesion(n=30)	
Grey	8(27%)
Brown	11(37%)
Black	10(33%)
Red	1(3%)
Ulceration(n=22)	
Yes	15(68%)
No	7(32%)
Pathological diagnosis of preoperative biopsy(n=27)	
Yes	23(85%)
No	4(15%)
Clinical Stage (FIGO)(n=27)	
I	1(4%)
II	7(26%)
III	13(48%)
IV	6(22%)
Satellitosis(n=30)	
Yes	8(27%)
No	22(73%)
Metastasis(n=10)	
Lung	3(30%)
Lymph nodes	7(70%)
Treatment(n=27)	
S alone	3(11%)
IT alone	1(4%)
S + IT	16(59%)
S+TT+CT	1(4%)
S + IT + CT	3(11%)
IT+TT+CT	1(4%)
S + IT + CT + RT	1(4%)
S + IT + CT + TT	1(4%)
Recurrence(n=27)	
Yes	18/27(67%)
No	9/27(33%)
Recurrent Site(n=18)	
Local	4/18(22%)
Other	14/18(78%)

S, Surgery; CT, Chemotherapy; RT, Radiotherapy; IT, Immunotherapy; TT, Targeted therapy.

### Pathological features

Among the 27 patients who underwent surgery, 23 (85%) were diagnosed with melanoma preoperatively. However, in two patients, a pathological examination of the cervical biopsy initially indicated malignant lymphoma and squamous cell carcinoma, whereas in two other patients, the vaginal biopsy result suggested poorly differentiated carcinoma and small round cell tumor. Postoperative immunohistochemical diagnoses confirmed all of them as melanomas. The immunohistochemical analysis shows yielding a positive rate of 93% for HMB-45 antigen, 90% for S-100, and 100% for Vim. In addition, 76% of the patients had a Ki-67 proliferation index greater than or equal to 30%. The rate of vascular infiltration was 14%. Among the 16 patients who underwent lymph node biopsy or lymph node dissection (LND), 63% were found to have lymph node (LN) metastasis. BRAF gene mutation testing was conducted in seven patients, with three of them showing positive results for BRAF V600E mutations (43%).

### Diagnosis and treatment process

Among the 27 hospitalized patients, 11 (41%) had melanoma located in the vulva, 14 (52%) in the vagina, and two (7%) in the cervix. Currently, a unified staging system for melanoma of the female reproductive system is lacking, and the staging standard recommended was different depending on the site. The American Joint Committee on Staging of Cancer (AJCC) recommends using the pTNM staging system for cutaneous melanoma as a reference for vulvar melanoma (5). The FIGO 2018 staging for cervical cancer is used as a reference for cervical melanoma pTNM staging (5). However, for vaginal melanoma, there is currently no specific AJCC staging system available. Given that vaginal melanoma was classified as mucosal melanoma, the literature tends to use the AJCC-TNM staging (6). In this study, the staging of vulvar melanoma was based on cutaneous melanoma TNM staging, cervical melanoma staging followed FIGO 2018 staging for cervical cancer, and vaginal melanoma staging followed TNM staging. Among the 27 hospitalized patients, one (4%) was in stage I, eight (30%) were in stage II, 13 (48%) were in stage III, and five (18%) were in stage IV. In addition, three (15%) patients already had lung metastases at the time of initial diagnosis.

Among the 25 patients who received surgery, the surgical treatment varied depending on the location of the melanoma. Patients with melanoma in the vulva underwent local extensive resection, sometimes accompanied by inguinal lymph node dissection (LND-I); those with melanoma in the vagina underwent local extensive resection or total vaginal resection, potentially with LND; and patients with melanoma in the cervix underwent extensive hysterectomy along with vaginal resection, often combined with pelvic lymph node dissection (LND-P). LND-P was performed for those with lesions located in the upper vaginal segment, and LND-I was performed for those with lesions located in the lower vaginal segment. Among patients who underwent LND, 10 underwent LND-P and eight underwent LND-I. In terms of treatment combinations, out of the 27 hospitalized patients, three underwent surgery alone, 16 underwent surgery combined with immunotherapy, three underwent surgery combined with immunotherapy and chemotherapy, one underwent surgery combined with chemotherapy and targeted therapy, one underwent surgery combined with immunotherapy, chemotherapy, and targeted therapy, and one underwent surgery combined with immunotherapy, chemotherapy, and radiotherapy. Moreover, two patients without surgery underwent immunotherapy, and one of them added chemotherapy and targeted therapy.

**Table 3** shows the clinical responses of 19 patients who had corresponding imaging data (chest, abdominal, and pelvic CT/MRI) before and after the initial treatment. After treatment, the overall complete remission rate of the 19 patients was 26%. For patients with vaginal and vulvar melanoma, the complete remission rates were 17% for vaginal melanoma patients and 40% for vulvar melanoma patients respectively. In addition, among the two cervical melanoma patients, one achieved complete remission. The overall objective response rate (ORR) was 42%, including an ORR of 42% for patients with vaginal melanoma and 40% for patients with vulvar melanoma. In the 17 patients who received immunotherapy, the complete response rate was 23%, and the ORR was 41%. In contrast, in the five patients treated with immune checkpoint inhibitors (like toripalimab and pembrolizumab), the complete response rate was 40%, and the ORR was 80%.

**Table 3:** Treatment response rate of female reproductive system melanoma patients (*n* = 19).

Characteristic of treatment response	Overall population (*n* = 19)	Vaginal melanoma (*n* = 12)	Vulvar melanoma (*n* = 5)	Cervical melanoma (*n* = 2)	Immunotherapy (*n* = 17)
CR, *n* (%)	5 (26%)	2 (17%)	2 (40%)	1 (50%)	4 (23%)
PR, *n* (%)	3 (16%)	3 (25%)	–	–	3 (18%)
PD, *n* (%)	11 (58%)	7 (58%)	3 (60%)	1 (50%)	10 (59%)
ORR, *n* (%)	8 (42%)	5 (42%)	2 (40%)	1 (50%)	7 (41%)

CR, complete response; PR, partial response; PD, progressive disease; ORR, CR + PR.

### Prognosis

#### Recurrence and metastasis

Among the 18 patients who experienced recurrence during the follow-up period, nine developed distant metastasis (lung, brain, liver, or bone), four had local recurrence, and nine had lymph node metastasis. The 2-year recurrence rate reached 82%. Both patients with cervical melanoma experienced recurrence. Among patients with vaginal melanoma, 10 out of 14 had recurrence, while six out of 11 patients with vulvar melanoma had recurrence.

#### Survival analysis

**Figure 1:**
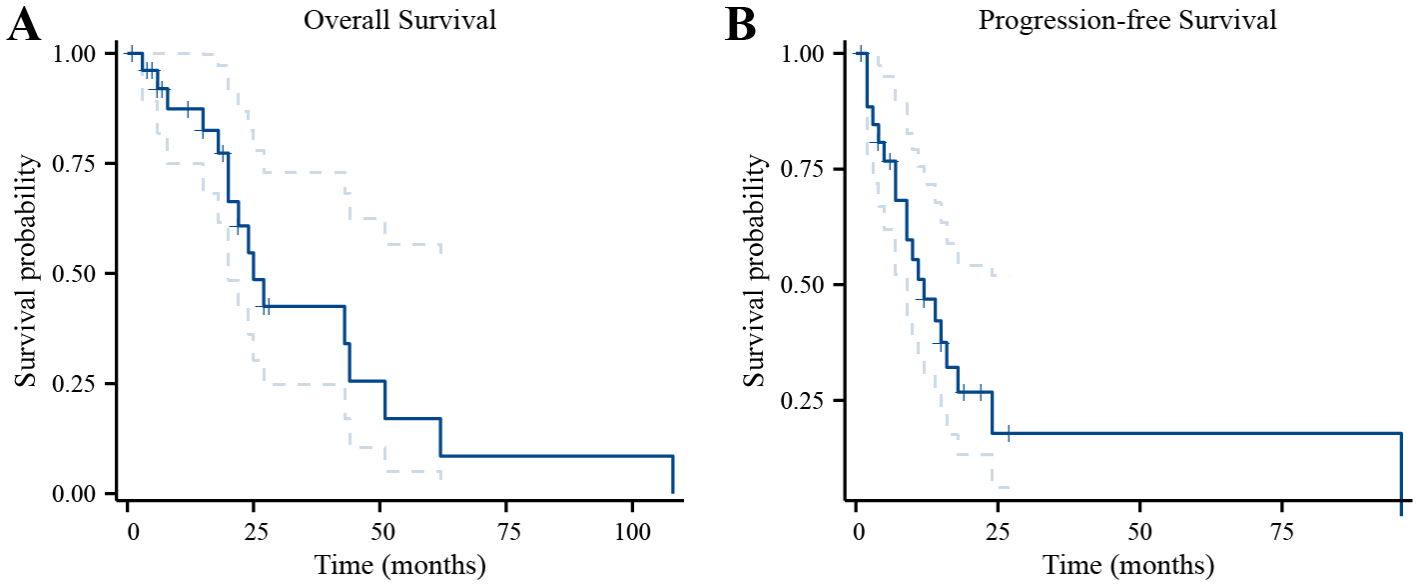
Survival curves of 27 patients with melanoma of the reproductive system. **(A)** Survival curves for the OS. **(B)** Survival curves for the PFS.

The OS and PFS curves of the 27 hospitalized patients are shown in **Figure 1**. Five patients were lost to follow-up. The OS rates at 1, 2, 3, and 5 years were 87%, 55%, 43%, and 17%, respectively. The 1- and 2-year PFS rates were 47% and 20%, respectively. The median PFS is 12 months, and the median OS is 25 months. Among the 27 patients, 10 had an overall survival period of 2 years or more. The clinical characteristics, diagnosis, and treatment of these patients are presented in **Table 4**. The OS rate, PFS rate, median PFS, and median OS of patients receiving immunotherapy are consistent with those mentioned above.

**Table 4:** Treatment of female reproductive system melanoma patients with OS ≥2 years (*n* = 10).

Case	Age	Location	Course	Tumor diameter	Stage	Surgical methods	Case	Initial diagnosis	Postoperative therapy	Recurrence (months)	Recurrent sites	Treatment after recurrence	OS (months)	Status
1	62	Vulva	12 months	4.5 cm	IIIC	Wide local excision of vulva + LND-I	1	2008	IFNα-2b + melanoma vaccine	12	Lung, liver, LN	IFNα-2b	24	DFD
2	34	Vagina	1 month	3 cm	III	Robot-assisted extensive total hysterectomy + total vaginal double salpingectomy + LND-P	2	2011	IFNα-1b + melanoma vaccine	7	Lung, bone, LN, pelvic metastasis	IFNα-1b + melanoma vaccine	43	DFD
3	73	Cervical	15 days	1 cm	IB1	Total hysterectomy + double adnexectomy	3	2011	IFNα-1b	96	Local	Toripalimab + IFNα-1b + vascular endothelial growth factor inhibitor	108	DFD
4	59	Vulva	20 days	1.7 cm	IV	Robot-assisted extensive vulvectomy + LND-I	4	2014	Local vulva radiotherapy + INFα-1b + melanoma vaccine + lung metastasis radiotherapy	11	Local, lung, ILN, stomach, muscles	Chemotherapy + multiple surgeries + IFNα-1b + toripalimab	62	DFD
5	63	Upper vagina	36 months	2 cm	III	Robot-assisted extensive hysterectomy + total vaginal + double appendages excision + LND-P + bilateral labia minora excision	5	2014	IFNα-1b + melanoma vaccine	14	LN	IFNα-1b + surgery	27	DFD
6	63	Lower vagina	20 days	0.5 cm	II	Robotic laparoscopic extensive total hysterectomy + total vaginal + double appendage resection + LND-P	6	2016	IFNα-1b	9	Local	Surgery + IFNα-1b	44	DFD
7	53	Upper vagina	10 days	2.2 cm	III	Laparoscopic extensive double appendage hysterectomy + total vaginal resection + LND-P	7	2018	IFNα-1b	2	Local, abdominal metastasis	Multiple surgeries + chemotherapy + pembrolizumab + tirelizumab + arotinib + bevacizumab	25	DFD
8	74	Lower vagina	50 days	0.8 cm	II	Extensive local excision of vaginal lesions	8	2018	IFNα-1b + pembrolizumab	24	Local, abdominal metastasis	Multiple surgeries + pembrolizumab + IFNα-1b + axitinib	51	DFD
9	59	Cervical	3 days	2 cm	IIIA	Laparoscopic extensive total hysterectomy + total vaginal + double appendage resection + LND-P	9	2021	Chemotherapy + carrelizumab	7	Local	Chemotherapy + pembrolizumab	28	AWD
10	56	Vagina	10 days	8 cm	IVB	Double adnexectomy + total vaginal resection + rectal resection + sigmoid colostomy + LND-P + LND-A + tumor cytoreductive surgery	10	2021	IFNα-1b + toripalimab	–	–	–	27	AWD

LND, lymph node dissection; LND-I, inguinal lymph node dissection; LND-P, pelvic lymph node dissection; LND-A, abdominal lymph node dissection; LN, lymph node; ILN, inguinal lymph node; PLN, pelvic lymph node; AWD, alive without disease; DFD, death from disease; DOC, death from other causes; LFFU, lost from follow-up.

**Figure 2:**
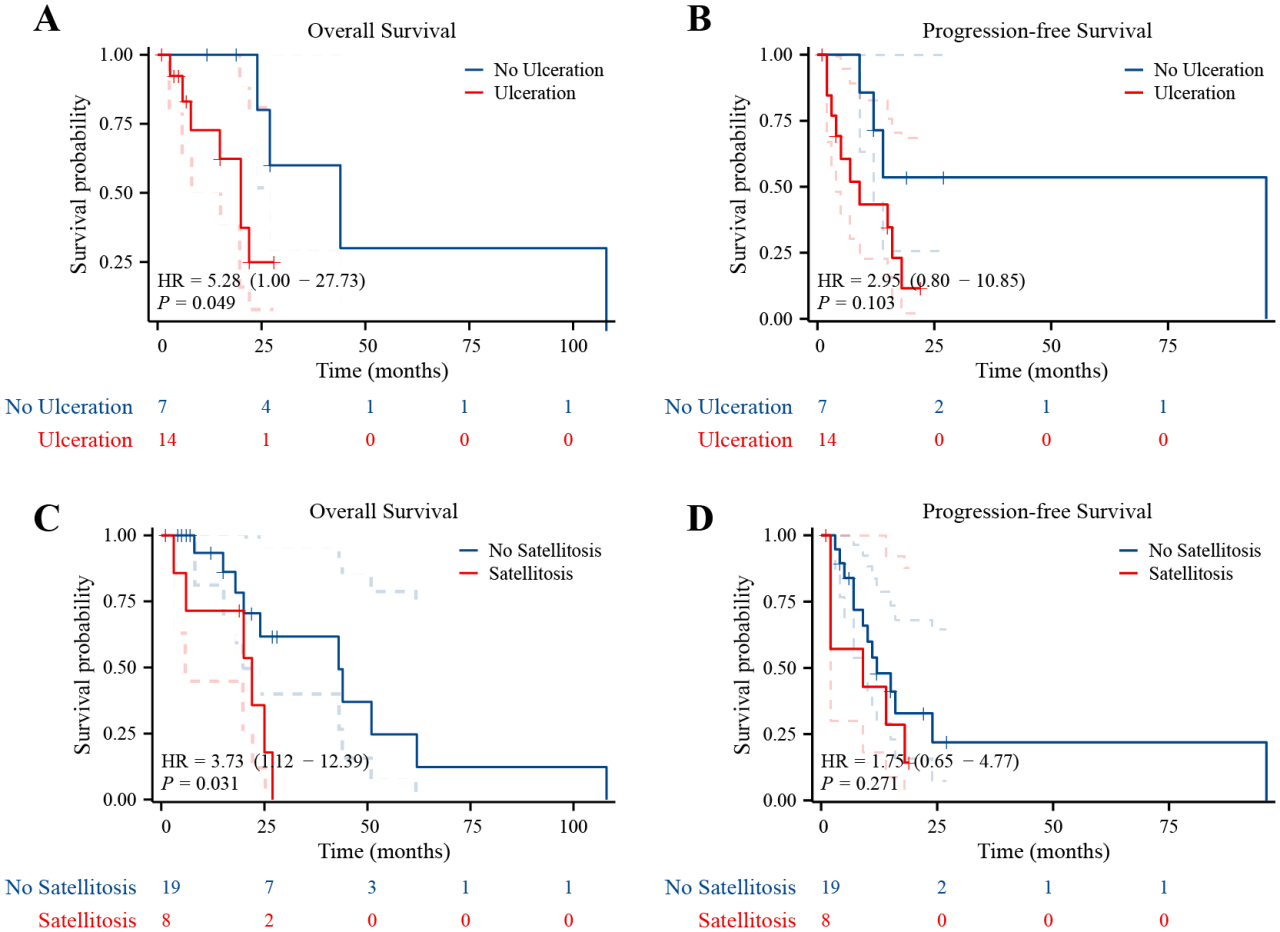
Ulcerated melanomas and satellite metastases had poor prognosis. **(A)** Kaplan–Meier survival curves for OS in patients whose lesions were with and without ulceration. **(B)** Kaplan–Meier survival curves for PFS in patients whose lesions were with and without ulceration. **(C)** Kaplan–Meier survival curves for OS in patients with and without satellite metastases. **(D)** Kaplan–Meier survival curves for PFS in patients with and without satellite metastases.

Due to the small number of cases, cervical melanoma survival analysis is not feasible. Survival analysis reveals no significant difference in overall survival (OS) or progression-free survival (PFS) between vaginal and vulvar melanoma (**Supplementary Figure S1**). In addition, survival analysis showed that patients with ulcerated melanomas had poor OS (*p* = 0.049) (**Figure 2A**), and patients with satellite metastases had poor OS (*p* = 0.031) (**Figure 2C**). It is worth noting that the PFS curves showed similar trends as the OS curves, but they were not statistically significant (**Figures 2B, D**).

## Discussion

Primary melanoma of the female reproductive system is a rare form of malignancy, accounting for 0.3%–1.3% of all melanomas and 2%–4% of malignancies of the female reproductive system (7, 8). The age of onset for this condition ranges from 14 to 90 years, with a peak incidence reported at 60–70 years (9). In this study, the age range of onset was 34–74 years, with a median age of 59 years. Notably, almost 80% of the patients in our study were postmenopausal, indicating that this condition is more likely to occur in elderly and postmenopausal women. These findings are consistent with previous research (5).

The most common initial symptoms of melanoma in the female reproductive system are abnormal bleeding and the presence of vulvar or vaginal masses. Among the 33 recorded patients in our study, 13 presented with abnormal bleeding, 18 had abnormal tumors, and one had abnormal vaginal discharge. The time from onset of symptoms to presentation varied, ranging from 3 days to 9 years. Of the diagnosed patients, 17 had a disease duration of less than 3 months, and 16 had a disease duration of greater than or equal to 3 months.

The primary sites of melanoma in the female reproductive system are the vulva and vagina, while occurrences in the cervix are rare, and those in the ovary are even rarer. It has been reported that ovarian melanoma mostly originates from ovarian mature teratomas (10). In our study, 45% of the primary melanoma involved the vulva, 49% involved the vagina, and 6% involved the cervix. No cases of ovarian melanoma were recorded.

The accurate diagnosis of melanoma of the female reproductive system depends on the biopsy pathology of the lesions. Any lesion with suspicious dermatoscopic, colposcopic, or clinical features should be subjected to biopsy. Melanoma is prone to misdiagnosis, especially in cases where the biopsy material is non-standard or when dealing with non-pigmented lesions. Immunohistochemistry plays an important role in confirming the pathological diagnosis of melanoma. S-100, HMB-45, and Vim are the commonly used immunohistochemical markers for diagnosing melanoma (11). In this study, the positive rate of HMB-45 was 93%, that of S-100 was 90%, and that of Vim was 100%. It is noteworthy that four (15%) patients were initially undiagnosed but were later confirmed to have melanoma through postoperative immunohistochemistry analysis. There were two patients with non-pigmented melanoma whose preoperative biopsies were misdiagnosed as poorly differentiated carcinoma and small round cell tumor, and two patients with lesion site at the cervix were misdiagnosed as malignant lymphoma and squamous cell carcinoma. However, their diagnoses were corrected to melanoma based on the results of postoperative immunohistochemistry. Hence, the accuracy of diagnosis of melanoma could be considerably improved through a combination of standard biopsy methods and immunohistochemical staining, enabling timely and appropriate patient management.

The rarity of this disease has conducted the lack of randomized studies. Current knowledge is based primarily on retrospective case series, case reports, and limited experience, often extrapolated from the treatment experience of skin melanoma or other gynecological cancers. There is currently no established guideline or clinical consensus on the staging of melanoma of the female reproductive system. In China, most gynecologists commonly refer to the FIGO staging of the lesion site for the diagnosis and treatment of this condition. The prognosis and tumor grading factors of vulvar melanoma are similar to those of cutaneous melanoma. The Breslow scale indicates the thickness and mitotic activity level, aiding in cutaneous melanoma staging (12). Chung’s classification, used in vulvar mucosal melanoma, has five levels to assess tumor invasion from the epithelium (5). The International Federation of Gynecology and Obstetrics (FIGO) staging for vulvar squamous cell carcinoma is a weak predictor of survival and treatment compared with micro-staging systems like Clark’s level, Breslow’s thickness, and Chung’s modified Clark system (13). The American Joint Committee on Cancer (AJCC) melanoma staging system is the most significant prognostic factor for vulvar melanoma. While thickness is a crucial melanoma indicator, FIGO 2018 staging system for cervical cancer, which is better related to the pattern of spread and prognosis, is widely used for cervical melanoma. Currently, no recommended staging system exists for vaginal melanoma (14). Due to its classification as mucosal melanoma, the literature tends to use the AJCC-TNM staging as the more relevant for prognosis (6). According to these staging standards, it was observed that 66% of the patients in this study were diagnosed at stage III–IV. This indicates that the majority of patients were diagnosed at an advanced stage, which could be one of the contributing factors to the poor prognosis of the disease.

The 5-year survival rate after complete resection of early cutaneous melanoma is more than 90%. The recurrence rate of mucosal melanoma after surgical treatment is 50% to 90% because it is often diagnosed at a late stage, with lymph node involvement, a complex anatomical location, and multifocal lesions (15). Neoadjuvant and adjuvant therapies can improve the postoperative outcomes of mucosal melanoma. The melanoma of the female reproductive system (cervix, vagina) mostly involves the mucosa. Although vulvar melanoma is an atypical cutaneous melanoma, the treatment principles and difficulties are similar to those of mucosal melanoma. The primary treatment approach for melanoma of the female reproductive system is surgery, supplemented by comprehensive treatment options such as interferon therapy, PD-1 inhibitors, and other immunotherapies used as adjuvant therapy after surgery. However, radiotherapy and chemotherapy are generally avoided owing to their limited efficacy in treating this condition. In recent years, there have been advancements in the selection of surgical methods and determining the scope of surgery, with an advocation toward reducing the extent of surgery (16).

The unique physiological structures of the vulva, vagina, and cervix, as well as their proximity to important structures such as the urethra, rectum, and anus, and the excessive surgical scope might bring serious complications. Studies have indicated that radical resection of vulvar cancer does not improve prognosis compared with extended local excision (17, 18). The extent of resection depends on the depth of tumor invasion. The recommended resection margins are as follows: for tumors with <1 mm invasion depth, resect at least 1 cm deep to the muscular fascia with 1 cm skin margins, 1–2 cm skin margins for 1–2 mm of invasion depth, and 2 cm skin margins for >2 mm of invasion depth (18). In this study, among the 11 patients with vulvar melanoma who were diagnosed and treated for the first time, five patients underwent radical resection, four patients underwent extended local excision, and two cases did not receive surgical treatment. In addition, five patients underwent LND-I, three of whom had lymph node metastasis and three had negative lymph nodes. The surgical wound of LND-I is large, and the benefits of LND-I for patients are still debatable. There have been studies suggesting that sentinel lymph node biopsy should be performed instead (19).

For vaginal melanomas, wide excision is considered an acceptable treatment. The recommended wide excision margins are consistent with vulvar melanoma: for tumors <1 mm, resect at least 1 cm deep to the muscular fascia with 1 cm skin margins, 1–2 cm skin margins for 1–2 mm thick, and 2 cm skin margins for >2 mm thick. Vaginectomy or pelvic exenteration are alternatives when wide excision is anatomically unfeasible, also for melanomas >3.00 mm in depth (18). Vaginal lymphatic drainage varies: the upper third drains to external iliac nodes, the middle third to common and external iliac nodes, and the lower third to superficial inguinal and perirectal nodes. SPECT-CT should be considered to assist with sentinel lymph node localization (18). A previous study did not find significant advantages of radical surgery compared with conservative local resection in terms of local control rate, disease progression-free survival, or OS in vaginal melanomas (20). In this study, among the 14 patients with vaginal melanoma who underwent surgical resection, 11 patients underwent radical surgery, which involved total vaginal resection and even pelvic exenteration. In addition to radical surgery, three patients underwent extensive local excision, and 10 patients underwent LND, seven of whom had lymph node metastasis and three had negative lymph nodes.

The primary surgical approach for cervical melanoma is radical hysterectomy with partial vaginectomy and LND-P. In cases where the lesion involves the lower vaginal segment, total vaginectomy along with LND-I may be performed (21). Pelvic lymphadenectomy aids in prognosis and planning adjuvant treatment (18). Among the reported cervical melanoma cases, patients treated with any type of surgery had a significant improvement in prognosis (22). The two patients with melanoma involved in the cervix in this study all underwent radical excision. One of them underwent LND and had negative lymph nodes.

In this study, five patients had melanoma lesions not only involving the vagina but also involving the cervix or vulva. Four of them underwent radical resection and LND, whereas one underwent extended local excision without LND. It is in this category of cases with a complex situation where individualized comprehensive treatment programs, including surgery selection, are needed. When the onset sites were multiple and difficult to determine, it is necessary to diagnose based on the primary lesion post-surgery as determined by pathological examination to guide the subsequent adjuvant treatment.

Interferon therapy is considered one of the effective treatments for melanoma of the female reproductive system, as it has been shown to prolong the OS and tumor-free survival of patients after surgery. Chemotherapy drugs, on the other hand, have low efficacy in the treatment of this condition. However, studies have suggested that chemotherapy combined with interferon therapy might exert a synergistic effect and enhance the treatment efficacy (23). In recent years, specific immunotherapy and targeted therapy have emerged as promising treatment options for melanomas, including that of the female reproductive system. In 2011, the cytotoxic T-lymphocyte-associated antigen-4 antibody ipilimumab was approved by the FDA for the treatment of advanced melanoma. This was followed by PD-1 monoclonal antibodies such as nivolumab and pembrolizumab (Nilum, Pyroram) and PD-L1 monoclonal antibodies, which have shown better efficacy and more controlled adverse reactions in the treatment of patients with advanced melanoma (24, 25). Targeted drugs, such as anlotinib and recombinant human endostatin, have shown efficacy in patients with recurrent melanoma (26). BRAF and NRAS mutations are common pathogenic mutations in melanoma. In cases with BRAF gene mutation, targeted therapy with BRAF and MEK inhibitors a potential treatment (27). For BRAF-mutated melanomas, the options include immunotherapy (nivolumab or pembrolizumab), or a combination of dabrafenib and trametinib may be considered (28). In general, in terms of clinical applications, interferon is one of the most commonly used treatments in the clinic. Nowadays, various comprehensive treatments combined could improve the survival and prognosis, including surgical treatment, postoperative radiotherapy, chemotherapy, immune therapy, or targeted therapy.

There were only seven patients who agreed to be tested for BRAF gene mutations in this study. One cervical melanoma patient was tested for a BRAF mutation and was found to be BRAF-mutated. Among the four tested vaginal melanoma patients, two had BRAF mutations. BRAF mutations were detected in up to 60% (3/5) of cervical or vaginal melanoma patients, which is inconsistent with the reported 10% activation of BRAF mutations in mucosal melanoma cases (22). The results were not representative because only five patients were tested for BRAF mutations in this study, but the detected BRAF mutations were of great significance to guide the subsequent treatment and predict the prognosis of these patients. Among them, a cervical melanoma patient with BRAF mutation had an earlier stage and did not receive postoperative adjuvant therapy. However, when vaginal stump recurrence occurred in the 8th year after surgery, she received immunotherapy and targeted therapy, achieving 108 months of survival. The other two BRAF mutation patients were both stage III vaginal melanomas and received corresponding immunotherapy after confirming the mutation. They relapsed at 2 and 6 months after surgery, and the final overall survival was 25 and 18 months, respectively. Genetic mutation testing is crucial for melanoma prognosis and treatment, but underutilized clinically, there are not many patients receiving the testing. Increased patient awareness is needed to facilitate better diagnosis and treatment decisions.

In a single-center, open-label phase II clinical trial, patients with mucosal melanoma received preoperative immunotherapy (toripalimab and axitinib) + surgery + postoperative immunotherapy (toripalimab), achieving an ORR of 33.3% (29). In another study, the patients received preoperative immunotherapy (lenvatinib and pembrolizumab) + surgery + postoperative immunotherapy (pembrolizumab), achieving an ORR of 40% (15). The POLARIS-01 studies reported that Chinese melanoma patients treated with toripalimab had an ORR of 17.3%, while the mucosal melanoma subgroup had an ORR of 0% (22/128) (30). Results from a phase II trial (HX008) showed that, as a second-line treatment, pucotenlimab had a lower ORR for mucosal melanoma compared with cutaneous melanoma (8.70% vs. 36.36%) (31). Moreover, immune checkpoint inhibitors had a less effective clinical response in Asians (ORR 0%–40%, PFS 1.4–14 months, OS 10.3–33.1 months) than in Caucasians (ORR 11.9%–23.3%, PFS 2.8–3.9 months, OS 11.3–15.97 months) (15). In a phase Ib study, toripalimab in combination with axitinib resulted in an ORR of 48.3%, a median PFS of 7.5 months, and a median OS of 20.7 months in patients with advanced mucosal melanoma (32). A phase II trial showed that in patients with advanced mucosal melanoma, the combination of atezolizumab and bevacizumab had a best ORR of 36.4% and a median PFS of 5.2 months (33). In a pooled analysis, nivolumab monotherapy was less effective in patients with mucosal melanoma (median PFS 6.2 months, ORR 40.9%) than in those with cutaneous melanoma (median PFS 3.0 months, ORR 23.3%). The combination of nivolumab and ipilimumab was less effective in patients with mucosal melanoma (median PFS 5.9 months, ORR 37.1%) compared with those with cutaneous melanoma (median PFS 11.7 months, ORR 60.4%) (34). In a single-arm study, the combination of camrelizumab and apatinib for advanced mucosal melanoma had an ORR of 42.9% and a median PFS of 8.05 months (35).

In the present study, 24 patients had received immunotherapies, and 22 of them received interferon therapy. Among the 24 patients who received immunotherapy, the complete response rate and objective response rate were 23% and 41%, respectively. The median PFS is 12 months, and the median OS is 25 months. This indicates that individualized and comprehensive treatment including immunotherapy can effectively increase the clinical benefits and improve the patients’ prognosis. Due to the lack of data on whole-body and cranial CT or MRI, we adjusted according to RECIST 1.1. Clinical response analysis was performed in patients with no extraperitoneal metastases of the lungs and pelvis at the initial treatment. The ORR of this study only reflected the changes in the abdomen/pelvic lesions and did not cover the brain and the whole body. Insufficient imaging coverage may result in an underestimation of disease progression. However, the pelvic and abdominal cavity is still the main metastasis site of the disease; the local efficacy has a certain reference value. New metastases or worsening symptoms in the unscanned area are regarded as “clinical progression”. By integrating clinical progress, we minimize bias as much as possible. Further prospective studies should include whole-body imaging to fully assess the efficacy.

Primary melanoma of the female reproductive system is characterized by a high degree of malignancy, poor prognosis, and a propensity for distant metastasis. Among the 27 patients who underwent treatment, three already had lung metastases at the initial diagnosis. The 2-year recurrence rate was high, up to 82%. With regard to recurrence and metastasis after surgery and comprehensive treatment, six patients had lung metastases, two had liver metastases, three had bone metastases, four experienced local recurrence, and nine had lymph node metastasis. These greatly impact the patients’ prognosis.

It has been reported that the 1-year OS rate for melanoma of the female reproductive system is approximately 80%, with a 5-year OS rate ranging from 25% to 30% (7). In this study, the 1-, 2-, 3-, and 5-year OS rate was 87%, 55%, 43%, and 17%, respectively, which was not completely consistent with other studies. The differences can be attributed to various factors, including the boundedness of the characteristics and staging of patients admitted to the hospital, the low incidence rate of melanoma in the female reproductive system, the lack of awareness among the local population regarding disease screening, and the economic constraints affecting subsequent diagnosis and treatment. In addition, high-risk factors such as ulceration and satellite metastasis impact survival in female reproductive system melanoma, which was confirmed in this study. The limited number and the large time span of cases, with evolving treatment methods and guidelines, make it difficult to analyze the survival groupwise across different treatments. This is a major challenge which is difficult to address in a rare disease clinical research.

## Conclusion

Primary melanoma of the female reproductive system is a rare and aggressive malignancy that significantly affects patient prognosis. Owing to its rarity and nonspecific clinical manifestations, early diagnosis is difficult, and the gold standard for diagnosis is pathological biopsy and immunohistochemical staining. Surgical resection remains the mainstay of treatment, with the extent of surgery depending on the tumor site and depth of tumor invasion. Comprehensive treatments such as immunotherapy and targeted therapy have shown promise in improving outcomes, which is expected to ameliorate the prognosis and improve the quality of life of the patients.

Although the retrospective, descriptive design, and limited number of cases of this study is not enough to form guiding opinions toward the diagnosis and treatment of this disease, the summary of clinical data on primary melanoma of the female reproductive system contributes to a better understanding of this disease and provides insights for further research and clinical practice. It underscores the importance of a multidisciplinary approach involving dermatology and gynecological oncology to enhance diagnostic accuracy, standardize clinical staging, and optimize treatment strategies in clinical practice.

## Data Availability

The original contributions presented in the study are included in the article/**Supplementary Material**. Further inquiries can be directed to the corresponding authors.

## Supplementary material

Supplementary Figure 1 Vaginal and vulvar melanoma prognoses are similar. (**A**) Kaplan–Meier survival curves for OS in vaginal and vulvar melanoma patients. (**B**) Kaplan–Meier survival curves for PFS in vaginal and vulvar melanoma patients.
