# Supplementary figures and images for: Diagnosis and treatment of 33 patients with primary melanoma of the female reproductive system
Source: Front Oncol. 2025 Aug 26;15:1615749. doi: 10.3389/fonc.2025.1615749 (PMC12417107; doi:10.3389/fonc.2025.1615749)

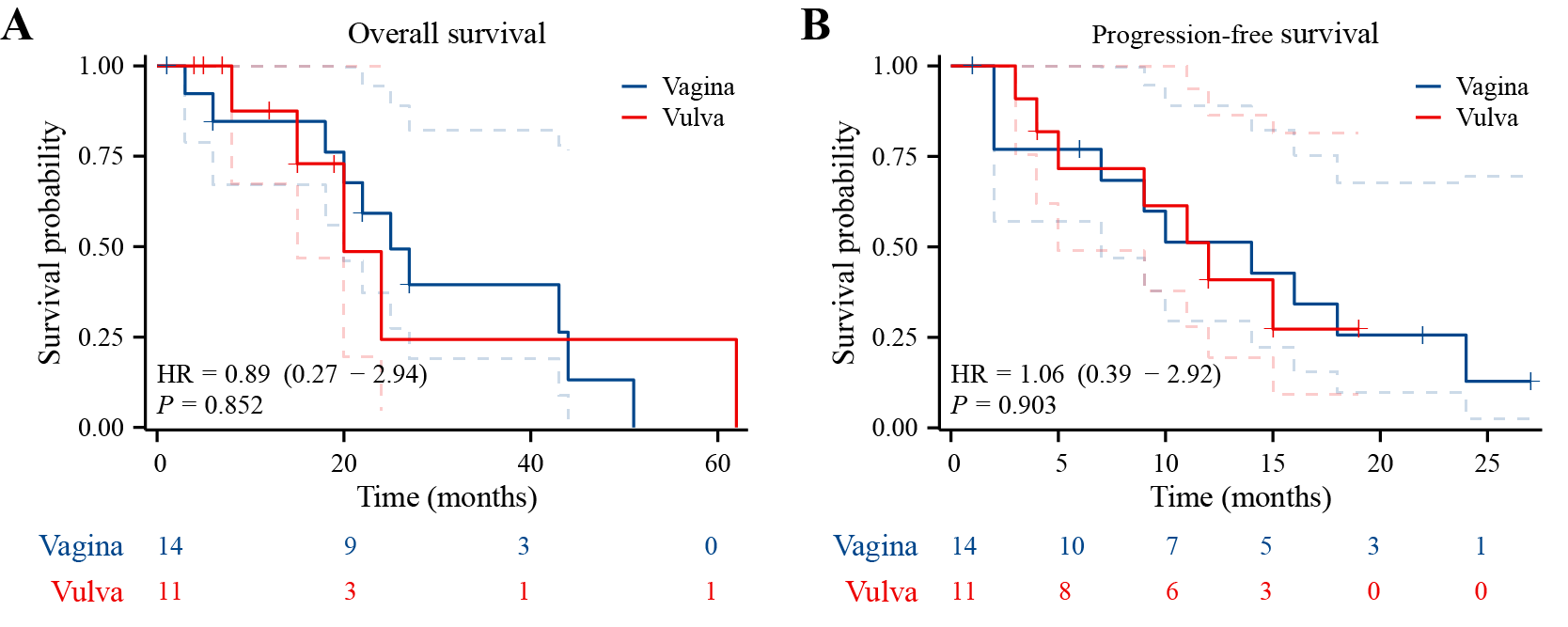

Supplement: Supplementary Figure 1 — Vaginal and vulvar melanoma prognoses are similar. (A) Kaplan–Meier survival curves for OS in vaginal and vulvar melanoma patients. (B) Kaplan–Meier survival curves for PFS in vaginal and vulvar melanoma patients. [file Image1.tif]
